# One-Stage Synthesis of Superhydrophobic SiO_2_ Particles for Struvite-Based Dry Powder Coating of Extinguishing Agent

**DOI:** 10.3390/nano15241859

**Published:** 2025-12-11

**Authors:** Igor Valtsifer, Yan Huo, Valery Zamashchikov, Artem Shamsutdinov, Ekaterina Saenko, Natalia Kondrashova, Anastasiia Averkina, Viktor Valtsifer

**Affiliations:** 1 Institute of Technical Chemistry, Ural Branch, Russian Academy of Sciences—Perm Federal Research Center, Ural Branch, Russian Academy of Sciences, 614013 Perm, Russia; 2 College of Aerospace and Civil Engineering, Harbin Engineering University, Harbin 150001, China; 3 Institute of Chemical Kinetics and Combustion, Siberian Branch, Russian Academy of Sciences, 630090 Novosibirsk, Russia

**Keywords:** spherical monodisperse particles, particles size, superhydrophobicity, contact angle, silicon dioxide, struvite, cohesion, flame inhibition

## Abstract

Methods for the one-stage superhydrophobic silica synthesis with high textural characteristics and a contact angle of up to 163° as a promising functional additive for struvite-based fire extinguishing powder compositions with complex gas-generating effects have been presented. In this work the sequence of components’ introduction into the reaction medium and the water amount influence during the one-stage synthesis of hydrophobic silicon dioxide on its textural, structural, morphological features and water-repellent properties were investigated. Rheological studies and assessment of the hydrophobic properties of fire extinguishing compositions, obtained with the synthesized silicon dioxide particles, allowed determining the optimal characteristics of a functional additive for compositions with struvite. The developed functional additive made it possible to implement the use of crystalline hydrates (struvite) in a fire extinguishing composition. Its inhibitory effect on flames is no less than two times greater than for widely used ammonium phosphates.

## 1. Introduction

Silicon dioxide is widely known for its various applications in a wide range of research. This is due to the role of silica in nature, its high chemical stability, as well as the presence of free surface silanol groups (Si-OH) capable of interacting with various inorganic and organic compounds, providing the desired functional properties of materials [1,2,3,4,5,6,7]. A great research interest is associated with the manufacture of silicon dioxide particles with a superhydrophobic surface, having a water contact angle exceeding 140°, which are widely used as a functional additive in fire extinguishing powder (FEP).

As a rule, FEP contains finely ground, easily decomposing inorganic salts (ammonium phosphates, sodium and potassium carbonates and bicarbonates) as an extinguishing component, and functional additives to increase the flowability and moisture resistance of powder compositions (nanodispersed particles of silicon dioxides and aluminum, magnesium stearate, talc) [8]. The technological barrier to the production of FEP is their caking tendency and high ability of the fine particles to aggregate leading to overall flowability of powders’ reduction [9]. It has been established that in most cases of long-term storage of powder compositions interparticle cohesion is the result of the capillary bonds occurrence between particles arising from the air moisture adsorption process [10]. As a consequence, amount of water incensement in the space between the powder particles leads to the growth of a film formed on their surface and formation of capillary bridges between the contacting particles.

One of the ways to minimize the negative effect of air humidity on powder compositions is to create a protective coating from nano-sized hydrophobic particles of a functional additive [11]. Investigations of V. Carde [12] and J. Elliott [13] revealed that, on the one hand, the coating can increase the distance and reduce the contact area between particles of the powder composition, decreasing the surface energy and the electrostatic charge. On the other hand, the coating particles have the capacity to absorb moisture and prevent the development of capillary bridges between the mixture particles.

Previously, our research group has developed methods for synthesizing functional fillers based on monodispersed spherical particles of silicon dioxide with superhydrophobic properties [14,15,16,17]. Introduction of these particles into fire extinguishing compositions in an amount of only 5 wt. % afforded free-flowing properties and a superhydrophobic surface state of FEP. It has been clearly shown that the created fire extinguishing powder composition based on monoammonium phosphate (MAP) and developed functional additives demonstrates the best specific efficiency of extinguishing solid and liquid flammable materials compared to analogs (Vekson ABC 50 EN (RF), Adex ABC (Poland), Glutex ABC (Germany)) used in powder fire extinguishing systems [18].

Improvement of FEP efficiency, depending on their purpose, can be achieved, by including substances with an inhibitory effect: potassium oxalate and alkali metal chlorides [19], salts of organic acids, for example, Trilon B [20], perfluoro (2-methyl-3-pentanone) [21], potassium iodide and potassium carbonate [22]; components with a cooling effect: mixture of aluminum hydroxide, aluminum oxide and activated aluminosilicate [23]; gas-generating components: urea [24], ammonium salts (chlorates, perchlorates, nitrates, acetates, formates, chlorides, oxalates, etc.) [25,26,27,28,29,30].

Recent developments of fire extinguishing agents with cooling and/or gas-generating ability for increasing the fire extinguishing efficiency by research groups from China covers implementation of “dry water”—water encapsulation by silica [31], dispersed hydrophobic magnesium hydroxide [32]; struvite (MgNH_4_PO_4_∙6H_2_O) [33]. Large focus is devoted to materials containing molecules with chemically bound water. It was shown that struvite is a particularly promising fire extinguishing compound due to its significant endothermic decomposition effect (~1400 J/g), environmental safety and ease of preparation [34].

However, a significant defect limiting the utilization of struvite in FEP is its tendency to undergo an intense recrystallization process during storage, which negatively affects the caking and flowability of the entire powder composition.

Several works are dedicated to the polymer protective coating on the surface of struvite particles and other fire extinguishing materials creation. Thereby the encapsulation method solved the problems of agglomeration, protection from air moisture and thermal stability, allowing the use of compositions at temperatures close to 100 °C [35,36,37,38,39].

Previously, we tested struvite as part of FEP of complex gas-generating effects by introducing a superhydrophobic functional additive based on silicon dioxide particles [40,41,42].

Herein we report a one-step method for the synthesis of the superhydrophobic functional additive for FEP which will significantly reduce the time and material costs for its preparation. The influence of the synthetic conditions for superhydrophobic silicon dioxide on its structural, textural and morphological properties was studied. Also, it was shown that struvite and superhydrophobic silicon dioxide-based FEP exhibits free-flowing properties. Moreover, we performed assessment of the inhibitory ability of a gas-generating FEP based on struvite and superhydrophobic silicon dioxide on the laminar burning velocity of an ethane-air mixture flow.

## 2. Materials and Methods

### 2.1. Materials Used for Synthesis

The following reagents were used to obtain hydrophobic silicon dioxide samples: tetraethoxysilane (TEOS, (Si(OC_2_H_5_)_4_, Sigma-Aldrich Chemie GmbH, Taufkirchen, Germany) as a source of silicon dioxide; polymethylhydrosiloxane (PMHS, (CH_3_(H)SiO)_n_, 99%, MM = 2500, Sigma-Aldrich Chemie GmbH, Taufkirchen, Germany) as a hydrophobizing compound; distilled water, ethanol (rectified) and ammonium hydroxide (NH_4_OH, 25%, analytical grade, AO Vecton, Saint-Petersburg, Russia) as a reaction medium.

The following reagents were used to prepare struvite: magnesium chloride hexahydrate (MgCl_2_∙6H_2_O, chemically pure, AO Vecton, Saint-Petersburg, Russia), ammonium hydrogen phosphate ((NH_4_)_2_HPO_4_, AO Vecton, Saint-Petersburg, Russia) and ammonium hydroxide (NH_4_OH, 25%, analytical grade, AO Vecton, Saint-Petersburg, Russia).

### 2.2. Synthesis Methods

In order to obtain silicon dioxide samples with different textural properties (particle size and specific surface area), the amount of water (mol) in relation to other synthesis components was varied according to the following ratios: TEOS/PMHS/NH_4_OH/C_2_H_5_OH/H_2_O-1:0.006:1.5:9.5:18-32.

Hydrophobic silica samples were prepared using two one-step synthesis methods:

- introduction of a hydrophobizing compound into the reaction mixture before the source of silicon dioxide (method 1, samples S1_1_–S5_1_);
- introduction of a hydrophobizing compound into the reaction mixture through a non-polar migration agent after a source of silicon dioxide and the formation of a SiO_2_-gel, (method 2, samples S1_2_–S5_2_).

In the case of samples S1_1_–S5_1_ (method 1) the calculated amount of PMHS was initially introduced into the reaction vessel with aqueous-alcohol solution which was then followed by an addition of ammonium hydroxide solution. After stirring, TEOS was slowly introduced into the reaction vessel, and the mixture was left until a dense gel formed. The contents of the reactor were transferred to an evaporation tank and kept in a drying cabinet at a temperature of 200 °C until dry; then, they were crushed.

Samples S1_2_–S5_2_ were prepared was as follows (method 2): an ammonia solution was added to a prepared aqueous-alcohol solution with pre-selected [H_2_O]/[C_2_H_5_OH] molar ratios and then a silicon source (TEOS) was added. PMHS solution in hexane (which is used here as a migration agent) was introduced to the resulting gel with the matrix solution while stirring. All the contents of the reactor were transferred to a rotary evaporator to remove the liquid phase, and then the residue was placed in a drying cabinet at a temperature of 200 °C for 2 h.

In the variants of the one-step synthesis of hydrophobic silica samples presented in this paper, we used the ratios of TEOS, H_2_O and C_2_H_5_OH that we adopted in a previous study. In that study, we produced hydrophobic SiO_2_ in two stages as follows: first, we made monodispersed silica (without PMHS) with various particle sizes (50–400 nm), and then we hydrophobized it with PMHS during prolonged boiling (for 4 h) in a non-polar solvent [39].

The fire extinguishing component synthesis—magnesium ammonium phosphate hexahydrate (struvite)—was carried out according to the following scheme: to solution of MgCl_2_∙6H_2_O 0.1 M and (NH_4_)_2_HPO_4_ 0.1 M an ammonia solution was added to pH 9.5. The mixture was stirred at 25 °C for 5 min. The precipitate was filtered off, washed with distilled water and dried at 25 °C for 24 h [33].

Powder mixtures were prepared for a comparative assessment of the rheological properties of FEPs based on struvite and MAP with hydrophobic silicon dioxide, where the amount of functional additive was 5 wt. %. The components were mixed in a ball mill for 3 h to obtain a uniform coating of functional additive on the surface of the extinguishing component [18].

### 2.3. Methods of Sample Characterization

Obtained samples were characterized by a wide range of physicochemical methods of analysis.

The sample surface modification by non-polar groups was studied by IR spectroscopy in the range 400–4000 cm^−1^ on an IFS-66/S FT-IR spectrometer (Bruker, Ettlingen, Germany).

Textural properties were determined by low-temperature nitrogen sorption on an ASAP 2020 instrument (Micromeritics, Norcross, GA, USA) after degassing the test material in vacuum at 90 °C for 3 h. The specific surface area of the samples (SBET) and the total pore volume (V_tot_) were determined by the BET method, distribution pores by size—by the BJH method using desorption isotherms.

The morphological properties of samples were studied by scanning electron microscopy on an FEI Quanta FEG650 instrument (Thermo Fisher Scientific, Eindhoven, The Netherlands).

The wetting angle of the surface of powder samples was estimated using a laboratory goniometer DSA100 (KRÜSS, Hamburg, Germany).

To assess the particle size distribution of powder compositions, a HELOS/KR laser diffraction analyzer (Sympatec GmbH, Clausthal-Zellerfeld, Germany) with a measurement range from 0.9 µm to 3.5 mm was used.

An FT4 powder rheometer (Freeman Technology, Tewkesbury, UK) was used to evaluate the rheological characteristics of FEP samples by performing shear and aeration tests [43]. Based on the results of shear tests under different normal loads on an FEP sample compacted under the same force, the cohesion and flow-function coefficient were determined. Cohesion reflects the influence of self-adhesion on the flow of coarsely dispersed materials; the flow-function coefficient qualitatively characterizes the powder flow regime [44]. Flow-function coefficient (*ffc*) is defined as the ratio of major principle stress to the unconfined yield strength and used as an indicator of powder flowability, that is, *ffc* < 1, “Not flowing”; 1 < *ffc* < 2, “Very cohesive”; 2 < *ffc* < 4, “Cohesive”; 4 < *ffc* < 10, “Easy flowing”; *ffc* > 10, “Free-flowing”.

The aeration cell of the FT4 was employed to evaluate the effect of aeration on the flow properties of the extinguishing powders. During dynamic flow, 48 mm-long curved blade passes through the powder layer along a spiral trajectory and induces forces that cause deformation and flow of the powder. The force parameters are measured continuously, in a way that the powder flow energy can be found. A 200 mL sample is placed in a cylindrical vessel with 50 mm diameter and is subjected to conditioning. Flow energy is measured at blade speed −100 mm/s; at these conditions air velocity can vary from 0 to 10 mm/s. Maximum air velocity corresponds to the Aerated Energy (AE).

**Figure 1:**
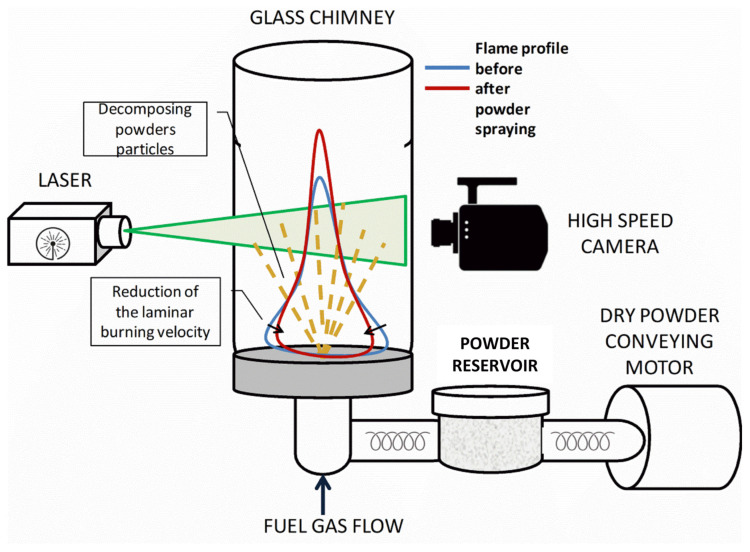
Instrument diagram for determining changes in the laminar burning velocity of an ethane-air mixture.

The assessment of the fire extinguishing ability of FEP was carried out using an instrument that allows one to determine changes in the laminar burning velocity while a powder composition is introduced into the flow of a combustible mixture (Figure 1). The main element of the installation is the burner—a quartz tube with 5 mm diameter. A pre-prepared gas combustible mixture of air with 5.7% C_2_H_6_ is supplied through the tube. A flame is installed at the end of the tube. A high-speed camera records the glow of the flame and FEP particles crossing the combustion front. The PIV (particle image velocimetry) complex allowed registering FEP particles in the flow both before and after the combustion front.

**Figure 2:**
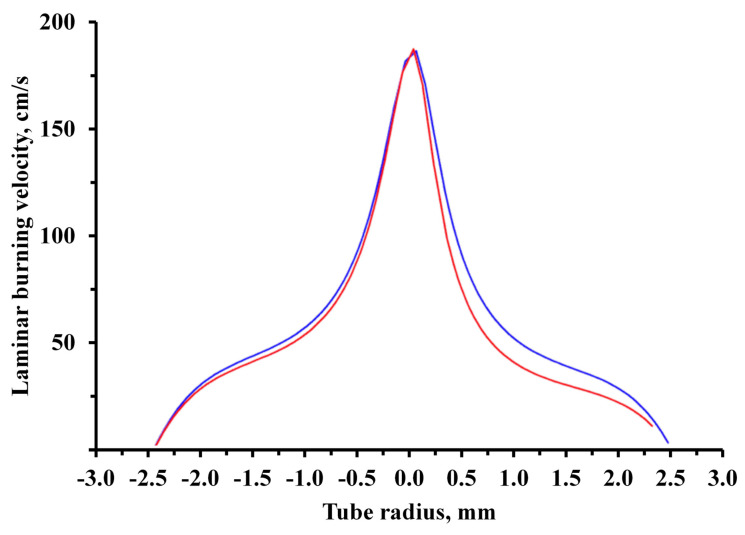
Dependence of the laminar burning velocity on the distance to the tube center without (blue line) and with (red line) the introduction of FEP particles.

The laminar burning velocity is defined as the speed of propagation of the combustion front relative to the initial mixture surface normal line. For saturated hydrocarbons the laminar burning velocity is about 30–50 cm/s. The dependence of the gas velocity flowing through a tube on the distance to its center *r* is expressed by the following formula:

(1) $$v=v_{0}\left(1-\frac{r^{2}}{R^{2}}\right)$$

where *v*_0_—velocity at the center, *R*—tube radius. Using the PIV complex, we demonstrated that this dependence is also valid for the velocity of the initial mixture inside the flame established at the end of the tube. The velocity *v*_0_ was determined using PIV and the flow rate of the initial mixture. A good data agreement was obtained. A normal line to a thin combustion front allows calculating the laminar burning velocity according to the formula:

(2) $$u_{n}=vcos\alpha ,$$

where α—angle between the normal line and the gas velocity vector. This correlation results from the immobility of combustion front: the combustion front tends to move along normal line inside the cone but is carried away by the gas flow. Introduction of FEP particles into the flow of a flammable gas mixture is followed by process of flame inhibition, leading to a decrease in the laminar burning velocity. In Figure 2, the blue line corresponds to the dependence of the laminar burning velocity on the distance to the tube center without FEP particles; the red line corresponds to the dependence with the introduction of FEP particles.

Due to heating of the initial mixture, the laminar burning velocity significantly increases in the center. For this reason, the effect of the inhibitor is compared on a flat section, where laminar burning velocity approximately corresponds to condition of normal temperature. The effectiveness comparison of FEP can be carried out by the difference measurements of the flame contour areas before and after the addition of a powder composition with a flame extinguishing effect (Supporting Information, Figures S1−S3).

## 3. Results and Discussion

### 3.1. Functional Additive Particles Characterization

To study the influence of the main components’ ratios during the synthesis of hydrophobic silicon dioxide by various methods on its textural and structural properties, two series of samples were prepared. Volume of the reaction medium was changed by increasing the amount of water (Table 1).

**Figure 3:**
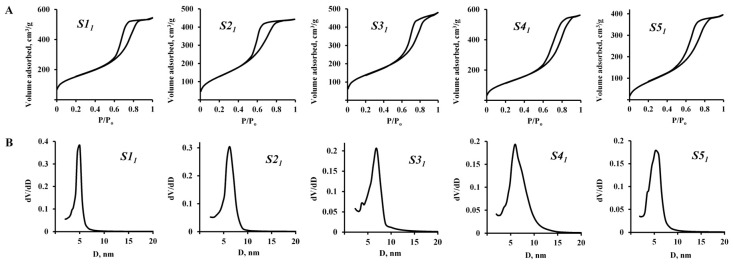
Sorption isotherms (**A**) and pore size distribution (**B**) in samples of hydrophobic silica obtained by Method 1.

**Figure 4:**
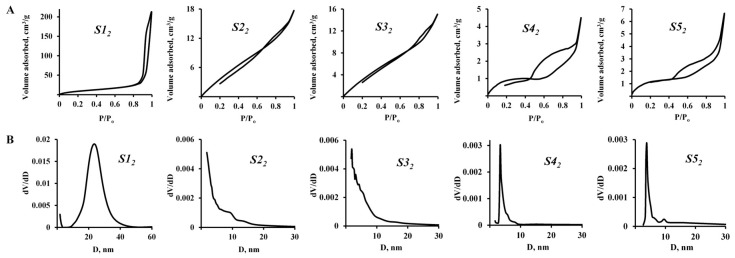
Sorption isotherms (**A**) and pore size distribution (**B**) for hydrophobic silicon dioxide samples obtained by Method 2.

Figure 3 and Figure 4, as well as Table 2, show the textural characteristics of hydrophobic silica samples obtained in the process of one-step synthesis using Methods 1 and 2.

The presented data reveal that the textural properties of hydrophobic functional additives obtained during one-step synthesis largely depend on the introduction sequence of the components into the reaction mixture.

In Figure 3, it can be observed that the shape of the nitrogen sorption-desorption isotherms of series 1 (S1_1_–S5_1_) samples, obtained by Method 1, with the introduction of a TEOS silicon source after the hydrophobizing compound (PMHS), corresponds to type IV (IUPAC), which is typical for mesoporous silica structures. The sorption isotherms hysteresis of this group of samples corresponds to the H2 type, which is referred to globular systems, including silica gels.

**Figure 5:**
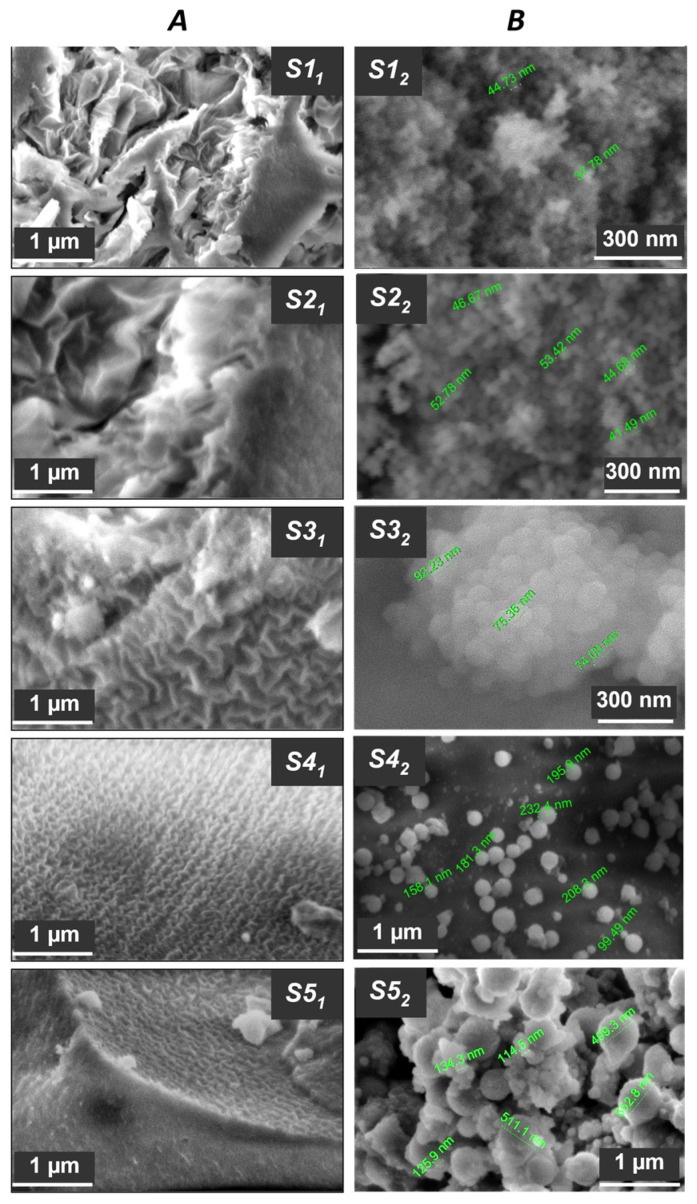
SEM images of hydrophobic silicon dioxide samples prepared by different methods: Method 1 (**A**), Method 2 (**B**).

Hydrophobic silicon dioxide samples obtained by Method 1 have a fairly high specific surface area (up to 512 m^2^/g), which slightly decreases through the lowering [H_2_O]/[TEOS] ratio (Table 3). The distribution of pores in samples of this group is determined in values up to 15 nm. Also, it can be assumed that the hydrophobizing compound PMHS, having in its composition both non-polar (Si–C, Si–H) and polar (Si–O–Si) functional groups and bearing the tensioactive properties, is playing the role of a structure-forming agent in this case. Thus, in Figure 5A a zigzag continuous channel-cellular structure of the hydrophobic silica samples surface-obtained by Method 1 has can be observed. Moreover, with the reduction in water content in the reaction medium during the synthesis of hydrophobic silicon dioxide by Method 1, the arrangement of cells becomes more ordered and their size decreases (Figure 3A, samples S3_1_–S5_1_).

Hydrophobic silicon dioxide samples S1_2_–S5_2_, obtained during a one-step synthesis by Method 2 (introduction of PMHS hexane is performed after TEOS) have lower textural characteristics. Textural properties, in this case, depend on the [H_2_O]/[TEOS] ratio (Table 3) and are determined by the morphological features of the samples. In Figure 5B, it can be observed that the samples of this group consist of spherical particles. Their size grows as the water content in the reaction mixture decreases from ~40 nm ([H_2_O]/[TEOS] = 32:1 (sample S1_2_) to ~500 nm ([H_2_O]/[TEOS] = 19:1 (sample S5_2_).

Figure 5 also reveals that samples of hydrophobic silicon dioxide S4_2_ and S5_2_, which were obtained by Method 2 using a minimum amount of water ([H_2_O]/[TEOS] = 19–22:1), are not monodisperse, unlike samples with ratios of main components which corresponded to the range [H_2_O]/[TEOS] = 25–32:1. This is especially visible in the SEM image of sample S5_2_, where two types of spherical particles with sizes of ~500 nm and ~100 nm are clearly identified.

This observation might be caused by TEOS hydrolysis rate slowing down with a small amount of water in the reaction medium in the presence of hexane, which is used as a migration agent to introduce the hydrophobizing compound PMHS into the composition. Preparation of samples S1_2_–S3_2_ involved larger amount of water and TEOS hydrolysis was accompanied by condensation of silanol monomers which allowed the formation of monodisperse silicon dioxide particles with a size of ~40–100 nm.

Nitrogen sorption-desorption isotherms for samples obtained by Method 2 correspond to low-porosity materials, despite the fact that sample S1_2_ with a spherical particle size of ~40 nm has a rather high total pore volume (0.33 cm^3^/g), which most likely corresponds to the volume of interparticle distances. In this case, the average pore size (~25 nm) (Figure 5B—S1_2_) is comparable to the particle size (~20 nm) and the sorption isotherm hysteresis (Figure 5A—S1_2_) corresponds to type H1, which proves the presence of uniformly packed and similar-in-size globular particles. Sorption isotherms of samples S2_2_ and S3_2_ with a particle size of 50–100 nm characterize materials with a low degree of surface coverage, when adsorption proceeds according to Henry’s law and the adsorption amount is directly proportional to pressure. The sorption isotherms’ shape of samples S4_2_ and S5_2_ with large particles of different sizes (up to 600 nm) can be classified as type II according to the UPAC classification, which indicates the presence of mesopores with their narrow size distribution (Figure 5A,B). The sorption isotherms hysteresis of these samples corresponds to the H3 type, which indicates the presence of particles of heterogeneous size and slit-like pores.

The methods for obtaining hydrophobic silicon dioxide discussed in this article were proposed by the authors based on previous studies [39]. By comparing the particle sizes of SiO_2_ presented in the literature and silicon dioxide hydrophobized using Method 2 at the same ratios of the main components, we can note that the introduction of a migration agent into the reaction mixture leads to a decrease in particle size in samples S1_2_–S3_2_. These samples were prepared using ratios of [H2O]/[TEOS] of 32–25, while the particle size of these samples was 40–100 nm, compared with non-hydrophobic samples S1-0–S3-0, which had a particle size of 50–200 nm at the same [H2O]/[TEOS] ratios.

However, a small amount of water, [H2O]/[TEOS] = 19, was used to obtain a hydrophobic sample S5_2_ using Method 2. This led to an increase in the particle size to 500–550 nm compared to the non-hydrophobic S5–0 sample, as described in the literature.

**Figure 6:**
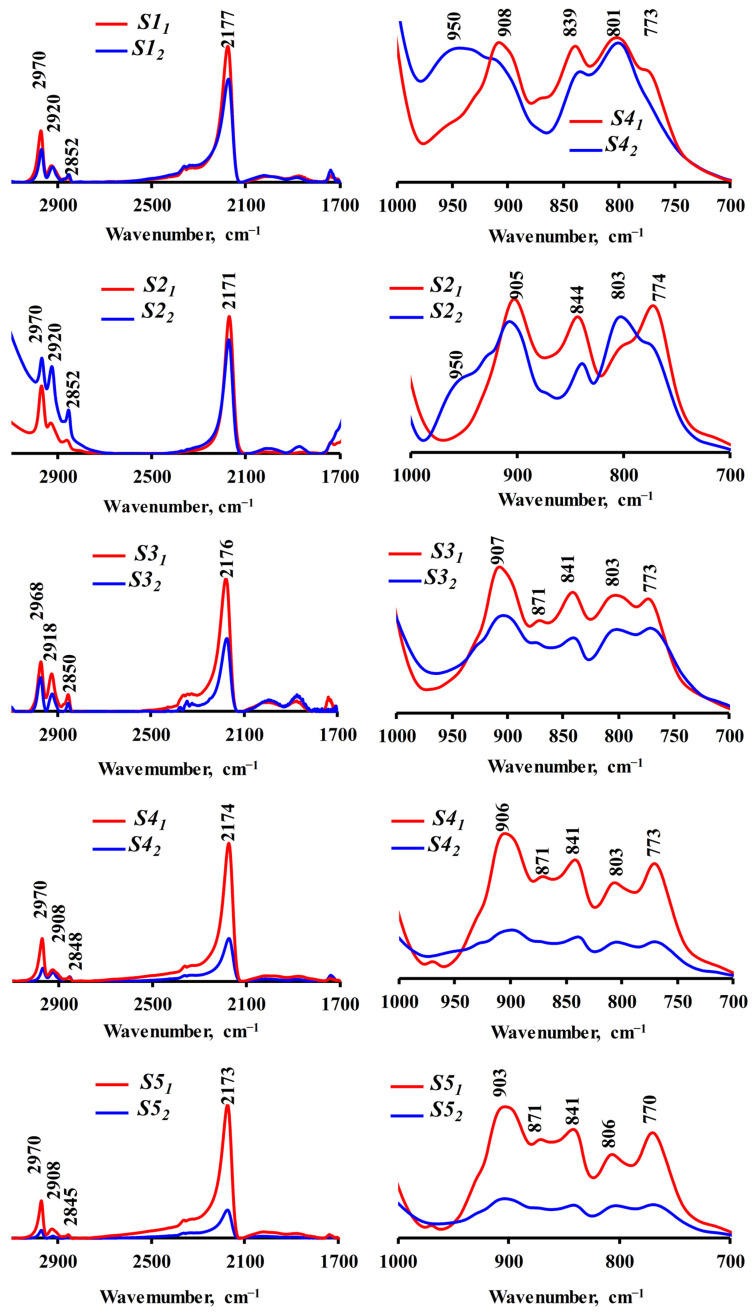
IR spectra of hydrophobic silicon dioxide samples obtained in the process of one-step synthesis by Methods 1 and 2 at different [H_2_O]/[TEOS] ratios.

IR spectroscopy data for hydrophobic silicon dioxide samples obtained by Methods 1 and 2 in a one-step synthesis at various [H_2_O]/[TEOS] ratios are shown in Figure 6. The IR spectra of all synthesized samples confirm the hydrophobization of the silicon dioxide surface by hydrophobic groups. Thus, absorption bands in the range 2980–2950 cm^−1^ correspond to symmetric and asymmetric stretching vibrations of the alkyl bond C–H; in the range 2171–2177 cm^−1^ correspond to the Si–H bond and at 903–908 cm^−1^; and 839–841 cm^−1^ can be attributed to vibrations of the Si–C bond.

The IR spectra demonstrate that in samples obtained by Method 2, the intensity of the characteristic absorption bands corresponding to nonpolar fragments decreases as the particles become larger. For the samples obtained by Method 1, the intensity of the characteristic absorption bands remains approximately at the same level, considering that the amount of the studied material in all cases was approximately the same.

Samples’ hydrophobicity study (Table 4) with a laboratory goniometer also demonstrates that reducing the amount of water and through the particles’ growth during process of one-step of hydrophobic silicon dioxide synthesis by Method 2, the contact angle decreases significantly. Since these samples are typical silica gels with a granule size of 1–4 mm, their use as functional additive is difficult. In this case, an additional grinding step is required.

### 3.2. Rheological Characteristics of Fire Extinguishing Compositions

Analysis results of the morphological, textural and hydrophobic properties of functional additives particles synthesized by various allowed selecting samples to create powder compositions and study their rheological properties. Compositions based on struvite FEPS-S1_1_, FEPS-S1_2_, FEPS-S3_2_, FEPS-S4_2_ were prepared. Samples with hydrophobic silicon dioxide S1_1_ and S1_2_ have the largest specific surface area and samples S3_2_ and S4_2_ were used to evaluate the particle size effect of the functional additive on the compositions’ flow. In order to comparatively characterize the properties of fire extinguishing powders, a composition based on MAP and FEPP-S1_2_ was additionally prepared. Here, specifically, the role of a functional additive is played by S1_2_ particles, which have a spherical shape and, at the same time, the highest values of specific surface area and contact angle among all the samples synthesized by Method 2.

**Figure 7:**
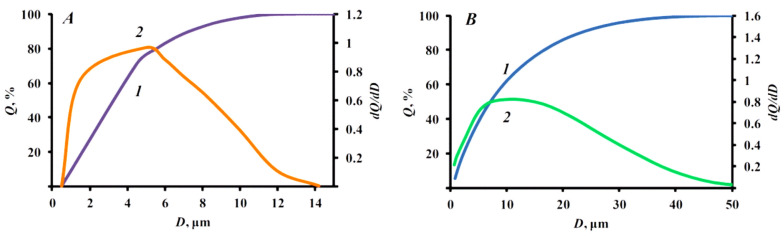
Particle size distribution of samples of FEP based on struvite (**A**), ammonium phosphate (**B**), where 1—integral distribution, 2—differential distribution.

Figure 7 compares the particle size distribution of FEP based on MAP and struvite. The introduction of functional additive into a powder composition means that the average size of struvite particles aggregates does not exceed 15 µm and ammonium phosphate—50 µm. In this case, the size of 50% of particle aggregates (D50) was 3.63 and 7.17 µm, respectively.

The formation of a protective coating of superhydrophobic silicon dioxide particles on the surface of struvite and MAP can significantly improve the water-repellent properties of substances compared to unmodified compounds. In this case, the surface of particles of samples of powder compositions is characterized by a wetting angle of more than 140° (Table 5).

**Figure 8:**
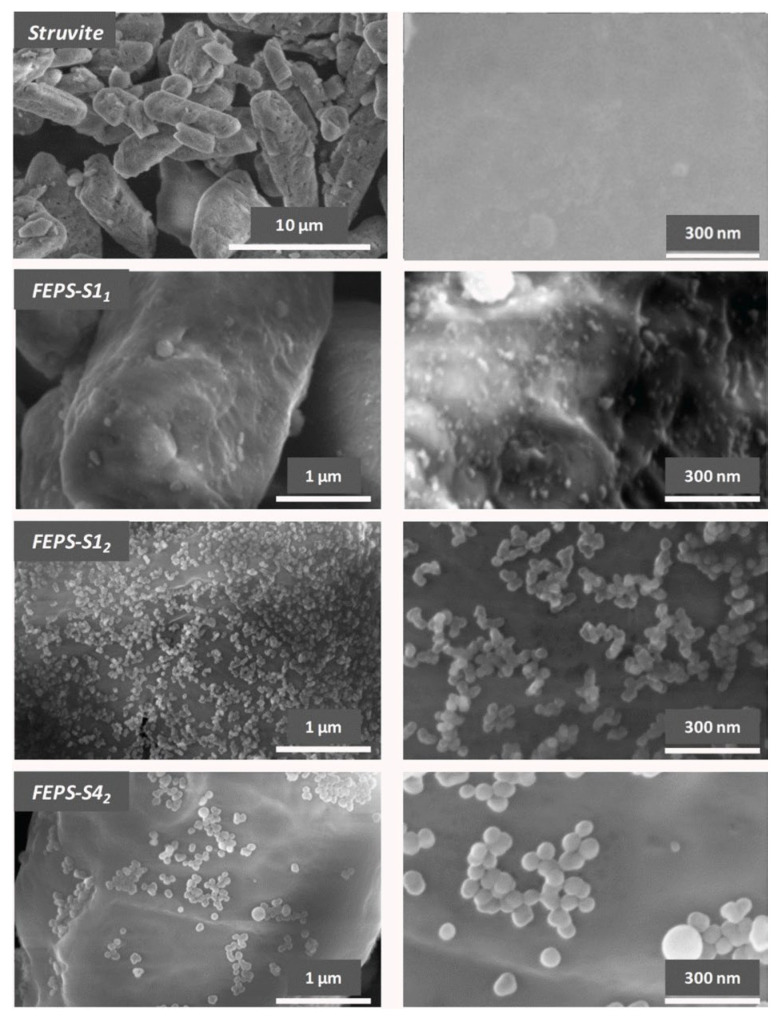
SEM images of fire extinguishing powder compositions.

However, the data given in Table 5 show that with the application of functional additive S1_1_ (prepared by Method 1) the struvite-based FEP sample (FEPS-S1_1_) remains hydrophilic. This, apparently, is due to the formation of large and strong agglomerates by the particles of S1_1_ sample, preventing the formation of hydrophobic coating on the surface of the extinguishing component particles (Figure 8).

**Figure 9:**
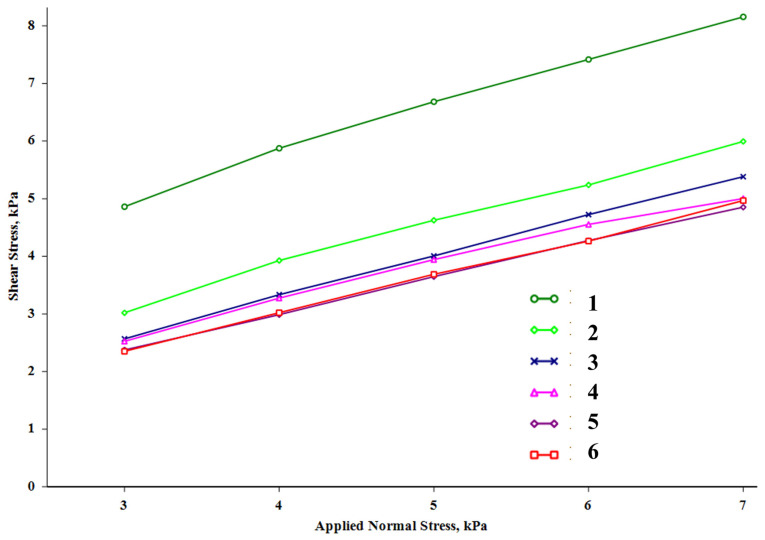
Shear test results of fire extinguishing powder compositions: (1) struvite; (2) FEPS-S1_1_; (3) FEPS-S1_2_; (4) FEPS-S3_2_; (5) FEPS-S4_2_; (6) FEPP-S1_2_.

SEM images comparison of fire extinguishing powder compositions FEPS-S1_2_, FEPS-S3_2_, FEPS-S4_2_ reveals that, with the particle diameter growth, the coating on the struvite surface becomes less uniform (Figure 8), leading to a number of contacts between particles and cohesion increasing (Table 6, Figure 9).

In our research, we found that the uniform distribution of the functional additive on the struvite surface has a crucial impact on the flowability of FEP samples (Table 6, Figure 9).

As it can be seen, with a decrease in the size of silica nanoparticles, the forces of interaction between them grow, which increases the strength of the agglomerates and makes it difficult to distribute nanoparticles over the surface of host particles. In the compositions of host particles, only if the nanoparticle volume percentage exceeds a certain value, the bulk cohesion reduces significantly; that means the formation of the uniform coating will happen [45]. Our research compared compositions with the same content of silicon dioxide nanoparticles in all samples. As the size of nanoparticles used in powder compositions increased, their amount became less sufficient to form a uniform coating. Furthermore, the difference in hydrophilicity of the silica nanoparticles affects how effectively their agglomerates break and then spread on the surface of the host particles [13].

Table 6 data show that the FEPS-S1_2_ composition (sample S1_2_ with a particle size of ~40 nm) is classified as free flowing (cohesion 0.420 kPa, *ffc* > 10) compared to pure struvite (cohesion 2.53, *ffc* < 2). Growth of the functional filler particle size, prepared by Method 2, to 200 nm (sample S4_2_) disrupts the uniformity of the coating and impedes the free flow of the composition (cohesion 0.932, *ffc* > 4).

**Figure 10:**
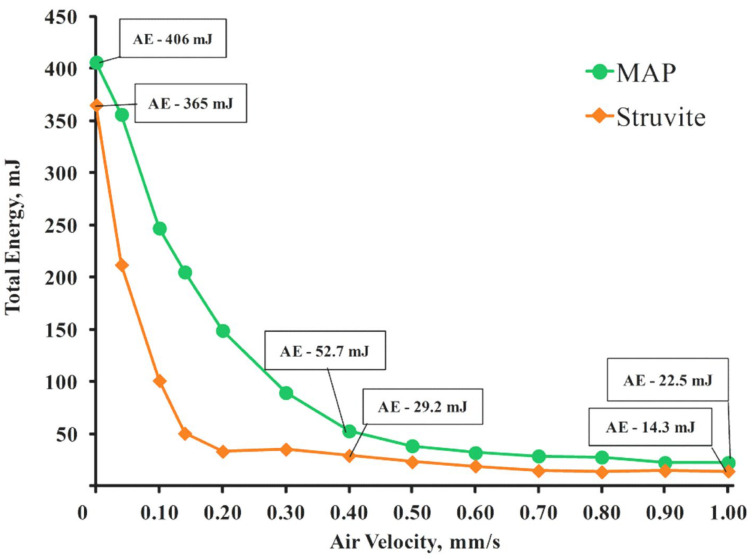
Dynamic characteristics of fire extinguishing powders based on MAP and struvite with the functional additive S1_2_ during the aeration test.

Aeration process assessment (Figure 10) allows correlating the influence of interparticle cohesion on the aeration energy values of the compositions; as a result the free-flowing fire extinguishing powder compositions FEPS-S1_1_ (based on struvite) and FEPP-S1_2_ (based on MAP) react differently to the speed of the air flow passing through layer of powder. The aeration energy for struvite particles coated with the S_1_ is 1.5 times lower than the aeration energy for MAP particles coated with the S_2_ (14.3 and 22.5 mJ at a flow rate of 1 mm/s).

In comparison with the results of studies on struvite-based FEP presented in the published literature [32,35,46], elaborated additive has the potential to significantly improve the flow and aeration of struvite, not only when compared to an untreated sample, but also when compared to a composition based on MAP. Moreover, a coating of silicon dioxide nanoparticles with a size of 40 nanometers on the surface of struvite, which does not require complex technological operations to obtain, is characterized by superhydrophobic properties with a wetting angle of approximately 160°.

### 3.3. Evaluation of the Compositions’ Ability to Inhibit Flames

The ability of powder compositions to inhibit flames was examined using the fire extinguishing powder compositions FEPS-S1_2_ (based on struvite) and FEPP-S1_2_ (based on MAP). A silicon dioxide S1_2_ sample with a particle size of 40 nm, prepared by Method 2, with the best hydrophobicity characteristics and the effectiveness of flow resistance reduction, was used for preparing samples of FEP.

Flame inhibition by FEP particles was assessed by the laminar burning velocity reduction in the ethane-air mixture, as well as by the number of particles passing on a flat section of the flame profile through an area of 1 mm^2^ in 1 s. Since the change in the laminar burning velocity becomes noticeable when the concentration of particles in the flow exceeds a certain critical value, a comparison of the critical limits for the number of particles of fire extinguishing powders allowed us to make a conclusion about the inhibitory effectiveness.

**Figure 11:**
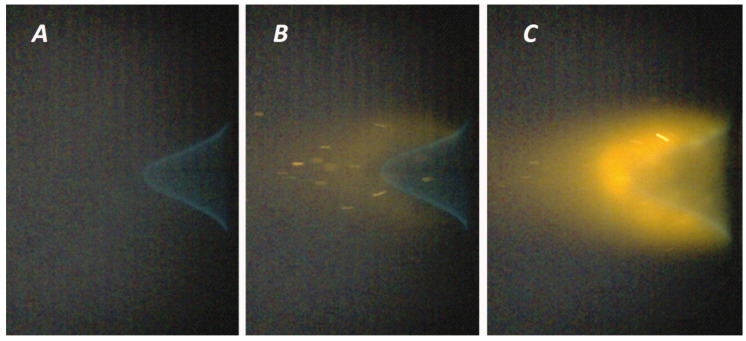
Images of the flame of an ethane-air mixture with the introduction of fire extinguishing powder particles ((**A**)—without particles; (**B**)—struvite particles; (**C**)—MAP particles) into the gas flow.

Figure 11 demonstrates the process of thermal destruction of the FEP particles in the flame of an ethane-air mixture. In this case, the one-stage process of thermal decomposition of struvite can be described according to the following scheme:

(3) $$2{MgNH}_{4}{PO}_{4}\cdot 6H_{2}O\to {Mg}_{2}P_{2}O_{7}+7H_{2}O↑+2{NH}_{3}↑$$

**Figure 12:**
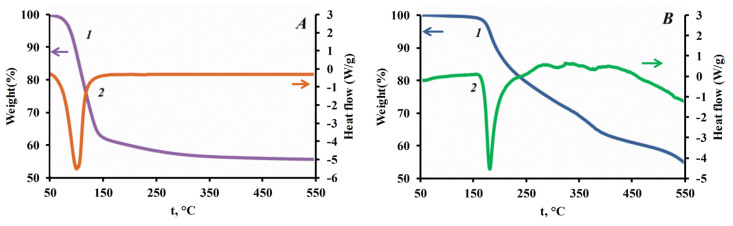
Results of thermal tests of the fire extinguishing powders samples (**A**) FEPS-S1_2_, (**B**) FEPP-S1_2_, where 1—TGA, 2—DSC.

While the thermal decomposition of MAP particles has several stages (Figure 12):

(4) $${NH}_{4}H_{2}{PO}_{4}\to {\left({NH}_{4}\right)}_{n-x}H_{x}{PO}_{3n-x}+H_{2}O+{NH}_{3}↑,200-400\;{}^{\circ}C,n=1-10^{6}$$

(5) $${NH}_{4}H_{2}{PO}_{4}\to {\left({NH}_{4}{PO}_{3}\right)}_{n}+H_{2}O,\geq 400\;{}^{\circ}C,n=1-10^{6}$$

A further increase in temperature above 450 °C leads to the loss of ammonia and the formation of an impermeable film of metaphosphoric acid:

(6) $${\left({NH}_{4}{PO}_{3}\right)}_{n}\to {\left(H{PO}_{3}\right)}_{n}+{NH}_{3},\geq 450\;{}^{\circ}C,n=1-10^{6}$$

The obtained TGA/DSC curves (Figure 12) confirm that the endothermic effects of struvite destruction in the temperature range ~50–194 °C are accompanied by heat absorption of about 1400 J/g (Figure 12A, Table 7). It is worth noting that the endothermic effect during the decomposition of MAP in the range 150–250 °C (Figure 12B Table 7) is significantly lower ~800 J/g.

It can be seen that during the thermal decomposition of struvite, most molecules enter the gas phase. In addition to water vapor, combustible ammonia is also formed. If we assume that the main mechanism of inhibition is dilution with an inert gas, then the particles of struvite powder should significantly reduce the laminar burning velocity compared to MAP powder. Also, when ammonia is added to a stoichiometric ethane-air mixture, oxygen deficiency occurs.

**Figure 13:**
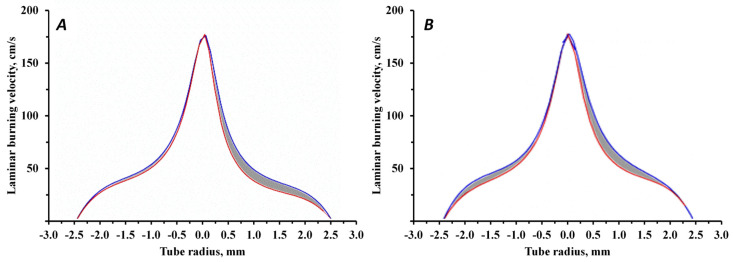
Laminar burning velocity without (blue line) and with (red line) the introduction of fire extinguishing powder particles based on MAP (**A**) and struvite (**B**).

Table 7 shows that the particle concentration limits required to achieve a similar inhibitory effect on the laminar burning velocity are very different for the two fire extinguishing compositions. It is obvious that the use of a struvite-based fire extinguishing composition made it possible to achieve the laminar burning velocity reduction by 26% (Figure 13) with half of the particle density (200 particles/mm^2^) than for MAP (450 particles/mm^2^ and the laminar burning velocity reduction by 29%).

Although two FEPs, based on MAP and struvite, have a similar chemical composition, they exhibit markedly different inhibitory effects when heated. When both powders are subjected to heat, water vapor and ammonia are released, indicating that their gas composition is similar. However, the difference in their inhibitory effects may be related to different heat adsorption during the decomposition. Moreover, struvite particles actively evaporate at lower temperature. As a result, the more particles begin to evaporate and the less heat is needed for evaporation, the greater their effect on the combustion process.

## 4. Conclusions

To sum up, in this work the conditions for the one-stage synthesis of superhydrophobic nanodispersed silicon dioxide were investigated. Its application as a protective coating for struvite particles made it possible to create a highly effective fire extinguishing agent.

Also, it has been determined that the introduction of the hydrophobizing compound PMHS into the reaction mixture before the TEOS allowed forming hydrophobic silica gels with a granule size of 1–4 mm, with a high specific surface area (up to >500 m^2^/g) and a narrow pore size distribution. The amount of water relative to the TEOS, in this case, does not have a significant influence on the properties of the synthesized samples. According to the results of the contact angle determination, all samples were superhydrophobic (contact angle > 150°).

Introduction of hydrophobizing compound PMHS into the reaction mixture by migration agent (hexane) after the TEOS, gave spherical, low porous particles the size of which increases with the water amount reduction used in the synthesis of hydrophobic SiO_2_. Hydrophobic silica samples with small particle sizes (40–100 nm) were superhydrophobic, with the wetting angle > 140°. The hydrophobicity of samples of this group decreased significantly with the [H2O]/[TEOS] ratio reduction.

We established that the use of 40 nm SiO_2_ (S1_2_) particles as a functional additive provided a protective coating on the surface of struvite particles with a contact angle of about 160°. The formed coating significantly reduces the cohesion (0.420 kPa) between crystalline hydrate particles, preventing their caking and imparting free-flowing properties to FEP (*ffc* > 10).

Evaluation of the inhibitory effect of FEP based on struvite and functional additive on the flame of an ethane-air mixture proved its effectiveness in comparison with a similar composition based on MAP. It was shown that to achieve the similar laminar burning velocity reduction efficiency, the density of struvite-based FEP particles is required to be half that of MAP-based FEP particles.

## Figures and Tables

**Table 1:** Molar ratios of components used in Method 1 and Method 2 for synthesis of SiO_2_ particles.

Molar Ratios					Introduction of PMHS into the Reaction Mixture			
TEOS	PMHS	NH_4_OH	C_2_H_5_OH	H_2_O	Before TEOS (Method 1)		After TEOS and the Formation of a SiO_2_-gel (Method 2)	
					Sample	Size, nm	Sample	Size, nm
1	0.006	1.5	9.5	32	S1_1_	Continuously porous structure	S1_2_	40
1	0.006	1.5	9.5	28	S2_1_		S2_2_	50
1	0.006	1.5	9.5	25	S3_1_		S3_2_	100
1	0.006	1.5	9.5	22	S4_1_		S4_2_	200
1	0.006	1.5	9.5	19	S5_1_		S5_2_	500

**Table 2:** Texture properties of SiO_2_ samples obtained by Method 1 and Method 2 with different [H_2_O]/[TEOS] ratios.

Method	Sample	H_2_O/Ethanol (mole) Ratio	SBET, m^2^/g	Vtot, cm^3^/g	Pore Diameter (BJH), nm	
					Adsorption	Desorption
1	S1_1_	32	512	0.69	4.7	4.4
	S2_1_	28	489	0.71	4.7	5.2
	S3_1_	25	481	0.74	5.7	6.0
	S4_1_	22	439	0.87	5.9	6.4
	S5_1_	19	349	0.61	5.3	4.9
2	S1_2_	32	37	0.33	24	26
	S2_2_	28	23	0.03	4.8	5.5
	S3_2_	25	20	0.02	5.7	4.8
	S4_2_	22	3	0.01	13.2	6.6
	S5_2_	19	3	0.01	15	9

**Table 3:** Textural characteristics of initial non-hydrophobic silicon dioxide samples [39].

Sample	H_2_O/TEOS	S_BET_, m^2^/g	S_micropores_ (t-Plot), m^2^/g	V_tot_, cm^3^/g	D_por_, nm	D, nm	Mass Loss 200–1000 °C, % (TGA)	Amount of Silanol Groups, mmol/g
S1-0	32	255 ± 5.6	44	0.97	14	50	3.7	4.11
S2-0	28	225 ± 7.6	134	0.37	20	120	3.9	4.33
S3-0	25	232 ± 8.4	151	0.26	18	200	4.1	4.56
S4-0	22	249 ± 9.4	190	0.17	6	300	4.3	4.78
S5-0	19	34 ± 0.26	27	0.05	8	400	4.5	5.00

**Table 4:** The value of the contact angle (θ, °) on the surface of hydrophobic SiO_2_ samples obtained in the process of one-step synthesis by Methods 1 and 2 with different [H_2_O]/[TEOS] ratios.

H_2_O/ TEOS (mole) Ratio	One-Step Method for Hydrophobic SiO_2_ Synthesis			
	Method 1		Method 2	
	Sample	Wetting Angle, °	Sample	Wetting Angle, °
32	S1_1_	153.2 ± 1.2	S1_2_	162.3 ± 0.3
28	S2_1_	152.8 ± 0.9	S2_2_	163.0 ± 1.2
25	S3_1_	163.4 ± 1.7	S3_2_	152.6 ± 0.8
22	S4_1_	162.1 ± 1.6	S4_2_	148.0 ± 1.4
19	S5_1_	155.8 ± 1.2	S5_2_	147.4 ± 1.4

**Table 5:** Contact angle on the surface of FEP samples.

Sample	Wetting Angle, °
FEPS-S1_1_	-
FEPS-S1_2_	160.7 ± 0.5
FEPS-S3_2_	152.8 ± 0.3
FEPS-S4_2_	148.3 ± 0.2
FEPP-S1_2_	160.2 ± 0.2

**Table 6:** Rheological characteristics of FEP based on struvite.

FEP Sample	Functional Additive	Cohesion, kPa	Flow Function Coefficient (*ffc*)
struvite	-	2.53 ± 0.05	1.75
FEPS-S1_1_	S1_1_	0.746 ± 0.04	5.97
FEPS-S1_2_	S1_2_	0.420 ± 0.02	10.70
FEPS-S3_2_	S3_2_	0.489 ± 0.06	9.07
FEPS-S4_2_	S4_2_	0.932 ± 0.03	4.47
FEPP-S1_2_	S1_2_	0.506 ± 0.05	8.30

**Table 7:** Assessment of the inhibitory ability of fire extinguishing powder compositions.

FEP Sample	Particle Density in Flame (Particles/mm^2^) in 1 s Interval	Laminar Burning Velocity Reduction, %	Heat Adsorption, J/g
FEPS-S1_2_	200	25.85	773.5
FEPP-S1_2_	450	29.07	1432.7

## Data Availability

All data are available upon request to the corresponding authors.

## Supplementary Materials

The following supporting information can be downloaded at https://www.mdpi.com/article/10.3390/nano15241859/s1, Figure S1: The flame propagation profile escaping from the tube; Figure S2: Dependence curve between the laminar burning velocity of flame and the distance to the center of the tube in the absence of particles of fire extinguishing powder; Figure S3: Dependence curve between the laminar burning velocity of flame and the distance to the center of the tube with addition of fire extinguishing powder particles.

## Abbreviations

The following abbreviations are used in this manuscript:

FEP	fire extinguishing powder
FEPS	fire extinguishing powder based on struvite
FEPS	fire extinguishing powder based on monoammonium phosphate
MAP	monoammonium phosphate
TEOS	tetraethoxysilane
PMHS	polymethylhydrosiloxane
