# Supplementary material for: One-Stage Synthesis of Superhydrophobic SiO2 Particles for Struvite-Based Dry Powder Coating of Extinguishing Agent
Source: Nanomaterials (Basel). 2025 Dec 11;15(24):1859. doi: 10.3390/nano15241859 (PMC12736022; doi:10.3390/nano15241859)
Supplement: Supplementary file 1 [file nanomaterials-15-01859-s001.zip › nanomaterials-4008807-supplementary.pdf]

Supporting information for

One-Stage Synthesis of Superhydrophobic SiO_2 Particles for Struvite-Based Dry Powder Coating of Extinguishing Agent

Igor Valtsifer [1], Yan Huo [2,*], Valery Zamashchicov [3], Artem Shamsutdinov [1], Ekaterina Saenko [1], Natalia Kondrashova [1], Anastasiia Averkina [1] and Viktor Valtsifer [1]

1 Institute of Technical Chemistry, Ural Branch, Russian Academy of Sciences—Perm Federal Research Center, Ural Branch, Russian Academy of Sciences, 614013 Perm, Russia

2 College of Aerospace and Civil Engineering, Harbin Engineering University, Harbin 150001, China

3 Institute of Chemical Kinetics and Combustion, Siberian Branch, Russian Academy of Sciences, 630090 Novosibirsk, Russia

* Correspondence: huoyan205@hotmail.com

Plotting a graph of the laminar burning velocity of an ethane-air mixture

The speed of gas flowing through a quartz tube depends on the distance *r* to its center as follows:

$$v = v_0\left(1 - \frac{r^2}{R^2}\right), \tag{S1}$$

where v_0 is the speed at the center of the tube and R is the radius of the tube. The speed profile does not change at a distance of several diameters from the tube (Figure S1). At least inside the flame, the speed is described with good accuracy by the proposed dependence.

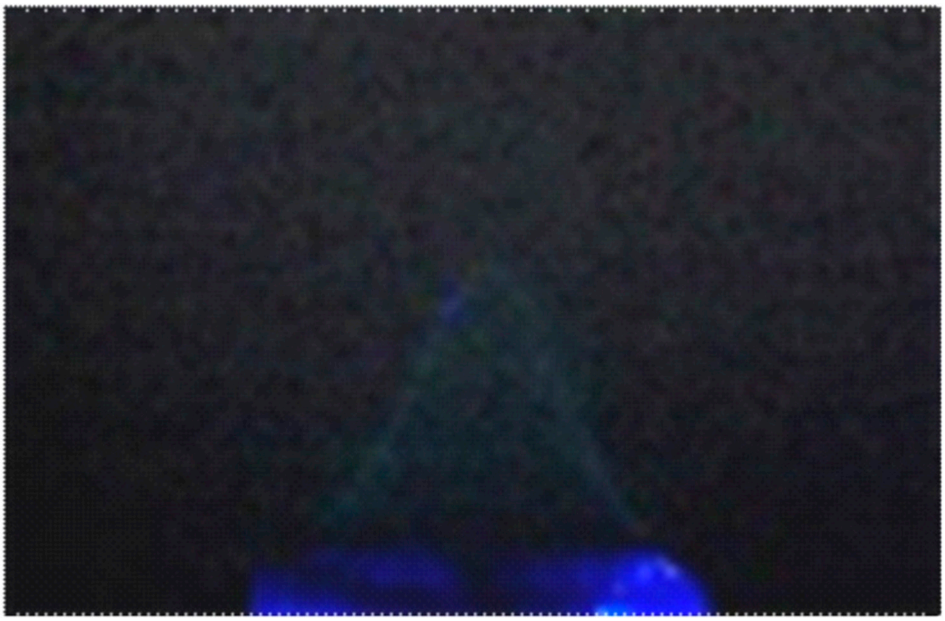

Figure S1 – The flame propagation profile escaping from the tube

The laminar burning velocity is the speed at which the flame moves relative to the original mixture normal to its surface. Since the flame is at rest, the normal flame speed must be equal to the projection of the gas speed onto the normal to the flame surface. Thus, the flame tends to move along the original mixture, and since the original mixture moves, it remains still. On the one hand, the normal speed near the tube wall decreases due to heat loss (heat flow into the wall). On the other hand, the flame speed increases in the middle of the tube because the source gas affected by diffusion into the side surfaces of flame changing its temperature and composition as it moves towards the top of the flame

The angle between the normal and the gas speed is calculated through the derivative. Then the projection of the gas speed onto the normal is calculated. The parameters are calculated in the OriginPro 2018 software. Dependence examples of the normal flame speed on the distance to the center of the tube for the cases of a pure ethane-air mixture and with the addition of fire extinguishing powder particles are presented in Figure S2 and Figure S3 correspondently.

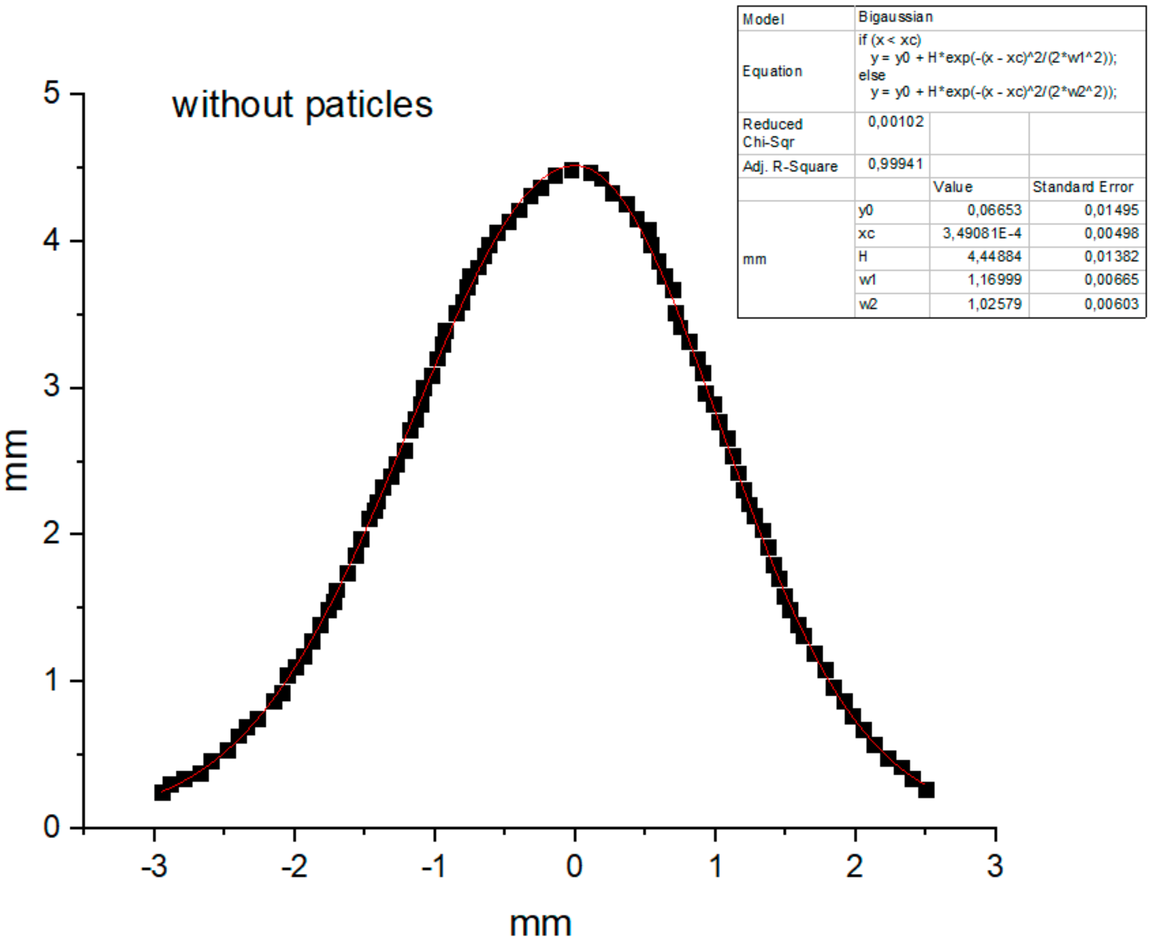

Model	Bigaussian		
Equation	if (x < xc)<br>y = y0 + H*exp(-(x - xc)^2/(2*w1^2));<br>else<br>y = y0 + H*exp(-(x - xc)^2/(2*w2^2));		
Reduced Chi-Sqr	0,00102		
Adj. R-Square	0,99941		
		Value	Standard Error
mm	y0	0,06653	0,01495
	xc	3,49081E-4	0,00498
	H	4,44884	0,01382
	w1	1,16999	0,00665
	w2	1,02579	0,00603

Figure S2 – Dependence curve between the laminar burning velocity of flame and the distance to the center of the tube in the absence of particles of fire extinguishing powder

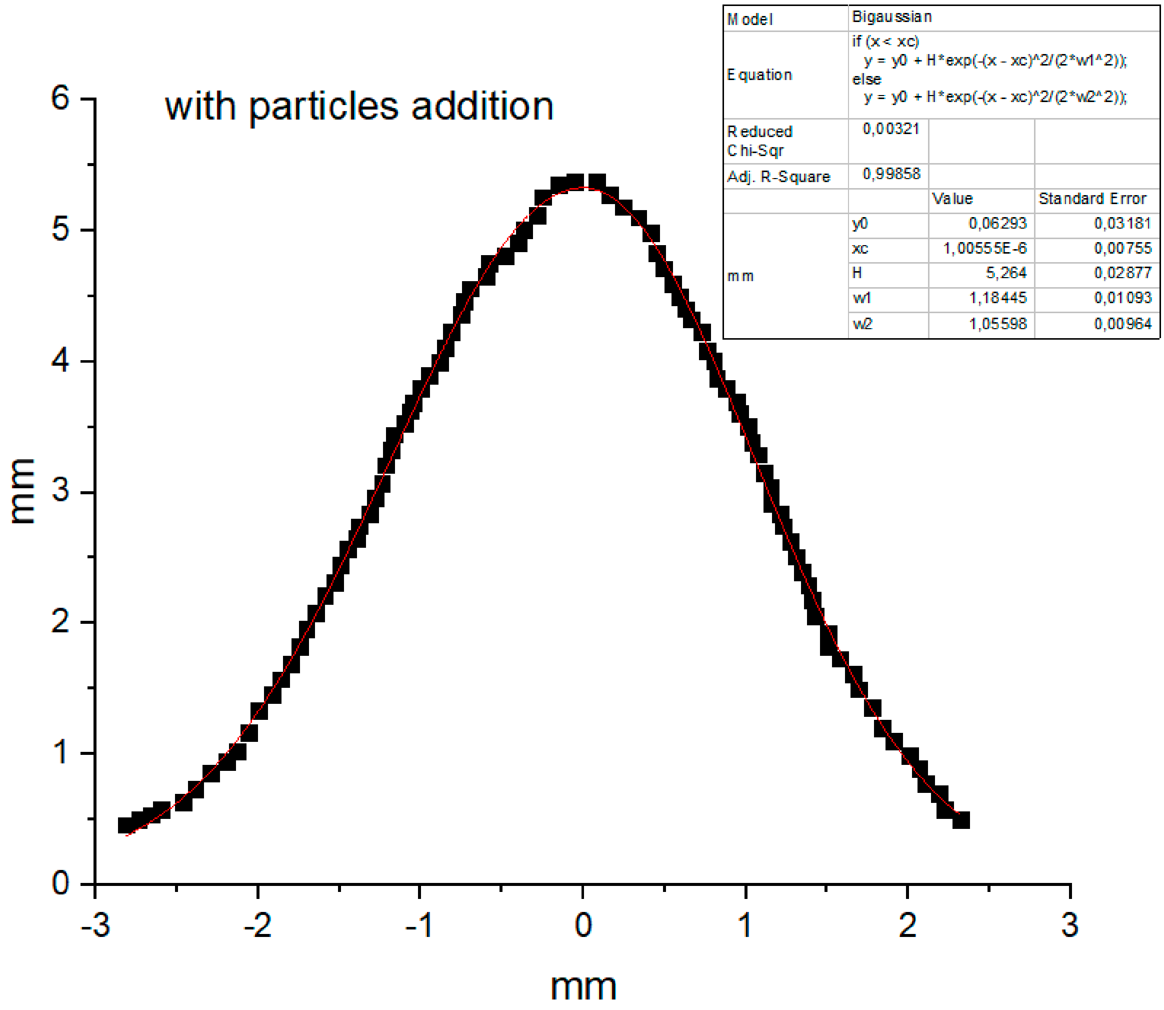

Figure S3 – Dependence curve between the laminar burning velocity of flame and the distance to the center of the tube with addition of fire extinguishing powder particles
